# Associations of vitamin D with coronary revascularization and heart rate variability in hypertensive patients

**DOI:** 10.3389/fcvm.2025.1590701

**Published:** 2025-09-19

**Authors:** Zongbin Li, Yuting Zou, Ruizhe Li, Minglei Zhang

**Affiliations:** ^1^ Department of Cardiology, The Sixth Medical Center of Chinese PLA General Hospital, Beijing, China; ^2^ Chinese PLA Medical School, Beijing, China; ^3^ School of Medicine, Nankai University, Tianjin, China

**Keywords:** hypertension, vitamin D, coronary revascularization, heart rate variability, aldosterone-to-renin ratio

## Abstract

**Background:**

Even though substantial evidence has found that vitamin D deficiency correlates to risk factors for cardiovascular disease (CVD), few studies have shown how vitamin D affects coronary revascularization and heart rate variability (HRV). We aimed to explore the connection between vitamin D levels with coronary revascularization and HRV in hypertensive patients.

**Methods:**

A total of the 250 eligible participants with hypertension hospitalized at the Department of Cardiology, Sixth Medical Center of Chinese PLA General Hospital was consecutively recruited. The status of vitamin D is measured utilizing serum 25-hydroxyvitamin D_3_ [25(OH)D_3_] concentrations. The primary endpoints were defined as patients undergoing coronary revascularization treatment. The secondary endpoints were defined as the variation in HRV. HRV indices were recorded in participants using a 24-h Holter electrocardiogram (ECG). In addition, direct renin concentrations and plasma aldosterone concentrations were measured in the supine and standing positions.

**Results:**

In the study, 165 eligible patients assigned to the vitamin D deficiency [25(OH)D_3_ < 20 ng/ml] group and 85 to the vitamin D non-deficiency[25(OH)D_3_ ≥ 20 ng/ml] group. In both univariate logistic regression analysis (OR: 2.46, 95% CI: 1.06–5.68; *P* = 0.036) and multivariate logistic regression analysis (OR: 2.54, 95% CI: 1.02–6.33; *P* = 0.046), the 25(OH)D_3_ < 20 ng/ml demonstrated to be a significant risk factor of primary endpoints for those hypertensive patients. Receiver operating characteristic curve (ROC) analysis showed the multivariable-adjusted model for predicting primary endpoints in patients with hypertension, with an area under the curve (AUC) of 0.73 (95% CI: 0.64–0.82, *p* < 0.001). Regarding secondary endpoints, the HRV indexes such as standard deviation of normal-to-normal (NN) intervals (SDNN) (*P* = 0.04), standard deviation of the averages of NN intervals in all 5-min segments (SDANN) (*P* = 0.03), and Triangle indexes values (*P* = 0.02) were significantly different in the two groups. Finally, we observed that hypertensive patients with vitamin D deficiency had significantly greater aldosterone and aldosterone-to-renin ratio (ARR) values than those having vitamin D non-deficiency.

**Conclusion:**

Vitamin D deficiency was prevalent in hypertensive patients and was independently associated with the risk of coronary revascularization. Vitamin D deficiency also affects HRV and ARR values in hypertensive patients.

## 1 Introduction

Vitamin D acts as a steroid hormone through the adhesion of its active form, 1,25(OH)_2_D, to the vitamin D receptor (VDR), which is discovered on various cells all over the body, such as cardiomyocytes, vascular smooth muscle, and endothelial cells (1–4). As a common nutritional disorder, vitamin D deficiency was very common in patients suffering from various cardiovascular diseases (CVD) (5–7). Serum vitamin D hormone deficiency has been reported to have a direct effect on the heart, is associated with increased cardiovascular disease and can independently modify cardiovascular risk (8–11). Despite the fact that low vitamin D levels have already been related to stroke, heart failure, and coronary artery disease (12), as well as the development of hypertension, their association with coronary revascularization in patients with hypertension is still unclear. Previous studies have demonstrated that vitamin D deficiency leads to structural and ion channel remodeling and cardiac autonomic nervous system (ANS) dysfunction (13, 14). Heart rate variability (HRV) is a promising non-invasive clinical tool for assessing autonomic nervous system regulation of the cardiac sinus node, capable of representing a qualitative and quantitative assessment of cardiovascular system homeostasis through ANS control (15–18). The study has found a correlation between lower 25-dihydroxy vitamin D [25(OH) D] status and decreased HRV in healthy populations (19), however, just a few investigations have explored the correlation among vitamin D and cardiac autonomic function in patients with hypertension. Renin plays an important role in blood pressure regulation, with experimental evidence that 1,25 dihydroxy vitamin D[1,25(OH)_2_D] inhibits renin synthesis in the kidney (20) and that vitamin D deficiency may stimulate the renin-angiotensin-aldosterone system (RAAS), increase angiotensin, atherosclerosis, and endothelial dysfunction, resulting in an increased risk of cardiovascular disease (21). A previous study found that normotensives from the general population aldosterone-to-renin ratio (ARR) possess a stronger predictive value for incident hypertension than renin or aldosterone alone (22). Another study demonstrated that lower 25(OH)D and 1,25(OH)_2_D concentrations are independently associated with a raised systemic renin-angiotensin system (23). However, few research has revealed whether vitamin D deficiency affects the value of direct renin, aldosterone concentration and ARR. Recent study has showed that vitamin D deficiency promoted atherosclerosis and was associated with an increase in coronary events in patients who have previously been diagnosed with coronary artery disease (CAD) (24–26). Coronary revascularization is a commonly used method for treating CAD which includes percutaneous coronary intervention (PCI), coronary artery bypass grafting (CABG), or percutaneous transluminal coronary angioplasty (PTCA) (27). However, the impact of vitamin D deficiency on coronary revascularization remains unclear. Consequently, we aimed to investigate the association of vitamin D levels with coronary revascularization and HRV in hypertensive patients in the present study. In addition, we also explored the impact of vitamin D deficiency on plasma direct renin, aldosterone, and ARR values in patients with hypertension.

## 2 Methods

### 2.1 Patient recruitment

A total of the 250 eligible participants with hypertension hospitalized at the Department of Cardiology, Sixth Medical Center of Chinese PLA General Hospital from April 2021 to April 2022 were consecutively recruited in this study. All enrolled patients had fasting blood drawn the day after being admitted to the hospital to test serum 25-hydroxyvitamin D_3_[25(OH)D_3_] levels. The participants were not included when there was a record of cardiac arrest, severe dyspnea, severe liver disease (including cirrhosis), renal failure (including dialysis), serious infection recently, and other granulomatous diseases. The medical ethics committee of the Chinese PLA General Hospital provided approval to the study's protocol, and the research was performed in compliance with the Declaration of Helsinki. All patients who participated in the study provided written informed consent.

### 2.2 Assessment of serum 25(OH)D_3_ concentrations

The main quantifiable and measurable form of vitamin D storage and transit in the blood, 25(OH)D_3_ is currently considered to be the most reliable indicator for determining vitamin D status (28). Serum 25(OH)D_3_ concentrations were measured in our study as an indicator of vitamin D levels *in vivo*. Electrochemiluminescence assay (Cobas e601, Roche, Geneva, Switzerland) was used to detect the concentration of serum 25(OH)D_3_. Following a long overnight fast (>12 h), blood was drawn the following morning. Standard operating procedures normally require 2 h to process blood samples. The Endocrine Society defines deficiency as levels of 25(OH)D less than 20 ng/ml (50 nmol/L) (29), therefore, we divided the enrolled hypertensive patients into the vitamin D deficiency group [25(OH)D_3_ < 20 ng/ml] and the vitamin D non-deficiency group [25(OH)D_3_ ≥ 20 ng/ml].

### 2.3 Aldosterone-to-renin ratio measurement

Blood samples were collected between 6:00 AM and 10:00 AM after an overnight fast. Blood is taken in the supine position in the early morning before rising, and the patient must wait four hours in the standing position after rising and moving around before further blood samples were taken. The automated chemiluminescent analyzer (LIAISON XL, DiaSorin S.p.A., Saluggia, Italy) was used to determine the plasma aldosterone concentration and the direct renin concentration from EDTA-plasma samples.

### 2.4 HRV indices

HRV was measured in compliance with the European Cardiac Society and the North American Society of Pacing and Electrophysiology guidelines (30). The HRV indices were derived from the 24 h Holter electrocardiogram (ECG) monitor recorded by CT−083S (Beneware Medical Equipment Co., Ltd., Hangzhou, China). HRV was measured using the Holter system software that comes with the device. We analyzed the following HRV indexes.: (1) SDNN, Standard deviation of normal-to-normal (NN) intervals; (2) SDNN index, Mean of the standard deviations of all NN intervals for all 5-min segments of the entire recording; (3) SDANN, Standard deviation of the averages of NN intervals in all 5-min segments of the entire recording; (4) RMSSD, The square root of the mean of the sum of the squares of differences between adjacent NN intervals; (5) PNN50, NN50 count divided by the total number of all NN intervals; (6)Triangle index, Total number of all NN intervals divided by the height of the histogram of all NN intervals measured on a discrete scale with bins of 1/12 8 s; (7) LF, the low- frequency band (in the 0.04–0.15 Hz frequency band); (8) HF, the high-frequency band (in the 0.15–0.4 Hz frequency band) (16, 30).

### 2.5 Study endpoints

The primary endpoints were defined as patients undergoing coronary revascularization treatment. The secondary endpoints were defined as the variation in HRV coronary revascularization treatment including both complete revascularization (CR) and incomplete revascularization (ICR). Time- and frequency-domain HRV analyses described the variation in HRV. In time-domain analysis, the 24-h Holter ECG record was used to calculate the SDNN, SDNN, SDANN, RMSSD, PNN50, and Triangular Index. LF, HF, and LF/HF parameters were additionally assessed by the 24-h Holter ECG record. Data on coronary revascularization treatments were obtained from medical records. Each recording was edited by a trained clinical physician.

### 2.6 Statistical analysis

A total of the 250 eligible participants who have been recruited in the research, we divided 165 patients into the vitamin D deficiency group [25(OH)D_3_ < 20 ng/ml] and the remaining 85 patients into the non-deficient vitamin D group [25(OH)D_3_ ≥ 20 ng/ml]. Continuous variables were expressed as mean ± SD or median [interquartile range (IQR)]. And categorical variables were expressed as *n* (%). The χ^2^ test was utilized for contrasting categorical variables. Normality was ascertained utilizing Kolmogorov–Smirnov test was performed for normality. For normally distributed continuous data, the *t*-test was used to compare between groups. The Mann–Whitney *U*-test was used to compare non-normally distributed continuous data between groups. The univariate and multivariate logistic regression analyses were used to explore the relationship of 25(OH)D_3_ levels with the primary endpoints. A receiver-operating characteristic (ROC) curve analysis was implemented to illustrate the efficiency of the model composed of 25(OH)D_3_ < 20 ng/ml, age, gender, prior PCI, and diabetes mellitus to distinguish between hypertensive patients with and without the primary endpoints. SPSS (version 22.0, IBM, Armonk, NY) and GraphPad Prism 8 software were used to carry out all analyses. All tests were two-tailed, with a 2-tailed *P*-value <0.05 considered significant.

## 3 Results

### 3.1 Baseline characteristics

The baseline characteristics of the trial participants were displayed in Table 1. Of the study cohort, 66.0% had 25(OH)D_3_ values deficiency [25(OH)D_3_ < 20 ng/ml] and an additional 34.0% had non-deficiency values [25(OH)D_3_ ≥ 20 ng/ml]. Individuals with 25(OH)D_3_ non-deficiency have a higher incidence of low hemoglobin (*P* < 0.001). Patients with 25(OH)D_3_ deficiency were more likely to be had a lower serum calcium concentration compared with patients who had 25(OH)D_3_ non-deficiency (*P* = 0.04). In addition, these patients had higher levels of parathyroid hormone (PTH). Patients with a 25(OH)D_3_ deficiency classified as 25(OH)D_3_ < 20 ng/ml had a median PTH value of 48 pg/ml (IQR: 38–64 pg/ml) vs. 39 pg/ml (IQR: 30–48 pg/ml) for individuals with 25 (OH)D_3_ non- deficiency (*P* < 0.001) (Table 1).

**Table 1:** Baseline characteristics of the study population.

Characteristics	25(OH)D_3_<20 ng/ml (*n* = 165)	25(OH)D_3_ ≥ 20 ng/ml (*n* = 85)	*P*-value
Age (years)	58 ± 16	57 ± 15	0.54
Male sex, *n* (%)	90 (54.6)	57 (67.1)	0.06
Smoking, *n* (%)	48 (29.1)	33 (22.4)	0.12
Drinking, *n* (%)	23 (13.9)	19 (29.1)	0.09
BMI, (mean ± SD) kg/m^2^	26 ± 3	26 ± 4	0.23
Diabetes mellitus	49 (29.7)	23 (27.1)	0.66
Hyperuricemia	41 (24.9)	19 (22.4)	0.66
Renal insufficiency	12 (7.3)	5 (5.9)	0.68
Prior PCI	15 (9.1)	7 (8.2)	0.82
Prior MI	8 (4.8)	2 (2.4)	0.34
Prior CABG	3 (1.8)	0	0.52
Hemoglobin, (mean ± SD) g/dl	134 ± 20	144 ± 18	**<0** **.** **001**
Platelet count	225 (181,260)	214 (181, 255)	0.35
LDH, (IQR) mg/dl	163 (143,181)	154 (141,170)	0.08
Total cholesterol (mean ± SD) mg/dl	4.2 ± 1.0	4.1 ± 1.1	0.93
Triglycerides, (IQR) mg/dl	1.5 (1.1, 2.0)	1.5 (1.0, 2.1)	0.94
HDL-cholesterol, (IQR) mg/dl	1.1 (0.9, 1.2)	1.0 (0.9, 1.2)	0.52
LDL-cholesterol (mean ± SD) mg/dl	2.5 ± 0.8	2.6 ± 0.9	0.64
Potassium, (mean ± SD) mg/dl	3.9 ± 0.6	3.7 ± 0.3	0.07
Calcium, (mean ± SD) mg/dl	2.28 ± 0.12	2.31 ± 0.10	**0**.**04**
Phosphate, (mean ± SD) mg/dl	1.22 ± 0.21	1.19 ± 0.19	0.28
PTH, (IQR) pg/ml	48 (38, 64)	39 (30, 48)	**<0** **.** **001**
LVEF, median (IQR) %	62 (58,65)	61 (59, 65)	0.28
Creatinine, median (IQR) μ mol/L	77 (66, 90)	77 (69, 94)	0.40

BMI, body mass index; CABG, coronary artery bypass grafting; HDL, high-density lipoprotein; LDH, lactic dehydrogenase; LDL, low-density lipoprotein; LVEF, left ventricular ejection fraction; MI, myocardial infarction; PCI, percutaneous coronary intervention; PTH, parathyroid hormone.

A *P*-value of <0.05 indicates statistical significance.

### 3.2 Primary endpoints

**Figure 1:**
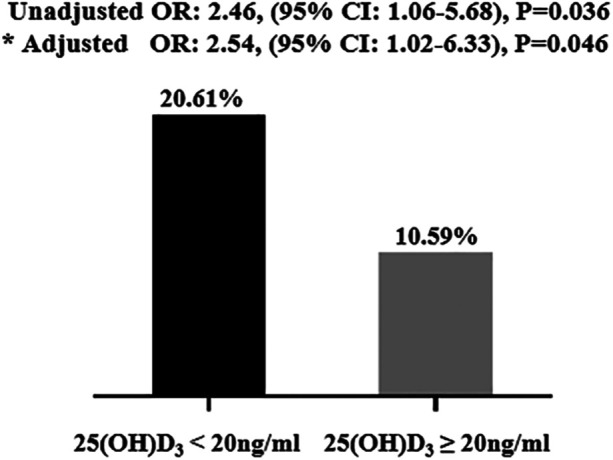
Clinical outcomes in the study population. The 25(OH)D_3_ < 20 ng/ml independently correlated with clinical outcomes. * Adjusted by age, gender, prior PCI, and diabetes mellitus. PCI, percutaneous coronary intervention.

**Figure 2:**
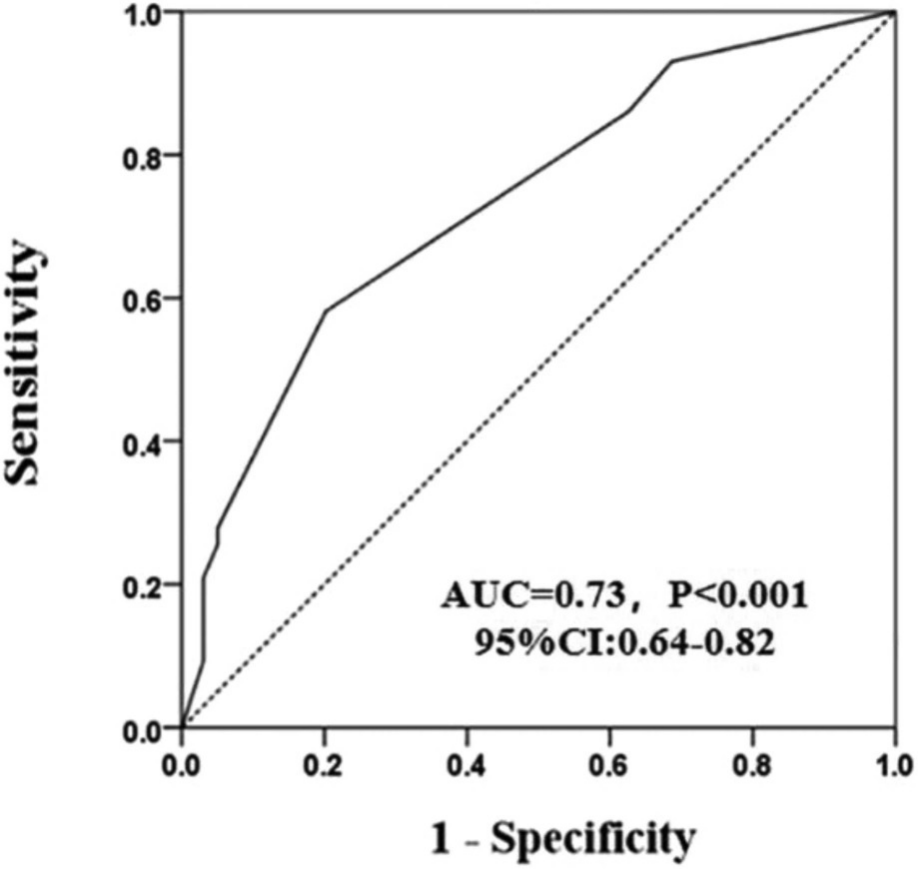
A receiver operating characteristic curves of the model composed of 25(OH)D_3_ < 20 ng/ml, age, gender, prior PCI, and diabetes mellitus.

The occurrence of coronary revascularization is connected to the 25(OH)D_3_ deficiency, 34 (20.6%) hypertensive patients undergoing coronary revascularization treatment in the 25(OH)D_3_ < 20 ng/ml group, and 9 (10.6%) occurred in the 25(OH)D_3_ ≥ 20 ng/ml group (*P* = 0.03) (Table 2). However, no relationship between 25(OH)D_3_ deficiency and the number of coronary vascular lesions were observed (Table 2). In univariate logistic regression analysis, a 25(OH)D_3_ < 20 ng/ml was associated with a higher risk of primary endpoints occurring in the hypertensive individuals (unadjusted OR: 2.46, 95% CI: 1.06–5.68; *p* = 0.036). Similarly, the 25(OH)D_3_ < 20 ng/ml was also an independent risk factor for those hypertensive patients in multivariable logistic regression analysis (adjusted OR: 2.54, 95% CI: 1.02–6.33; *p* = 0.046) (Figure 1). ROC analysis demonstrated that the multivariable-adjusted model [including 25(OH)D_3_ < 20 ng/ml, age, gender, prior PCI, and diabetes mellitus] identified hypertensive patients with or without coronary revascularization treatment [area under the curve (AUC): 0.73, 95% CI 0.64–0.82, *p* < 0.001] (Figure 2).

**Table 2:** The distribution of coronary vascular lesions.

Number of diseased vessels, *n* (%)	25(OH)D_3_ < 20 ng/ml *n* = 165)	25(OH)D_3_ ≥ 20 ng/ml (*n* = 85)	*P*-value
1 vessel	17 (10.3)	7 (8.2)	0.60
2 vessels	25 (15.2)	9 (10.6)	0.30
3 vessels	6 (3.6)	4 (4.7)	0.67
4 vessels and more	11 (6.7)	3 (3.5)	0.30
Coronary revascularization treatment^a^	34 (20.6)	9 (10.6)	**0** **.** **03**

CABG, coronary artery bypass grafting; PCI, percutaneous coronary intervention; PTCA, percutaneous transluminal coronary angioplasty.

A *P*-value of <0.05 indicates statistical significance.

^a^ Coronary revascularization treatment includes patients treated with PCI, CABG, and PTCA.

### 3.3 Secondary endpoints

**Figure 3:**
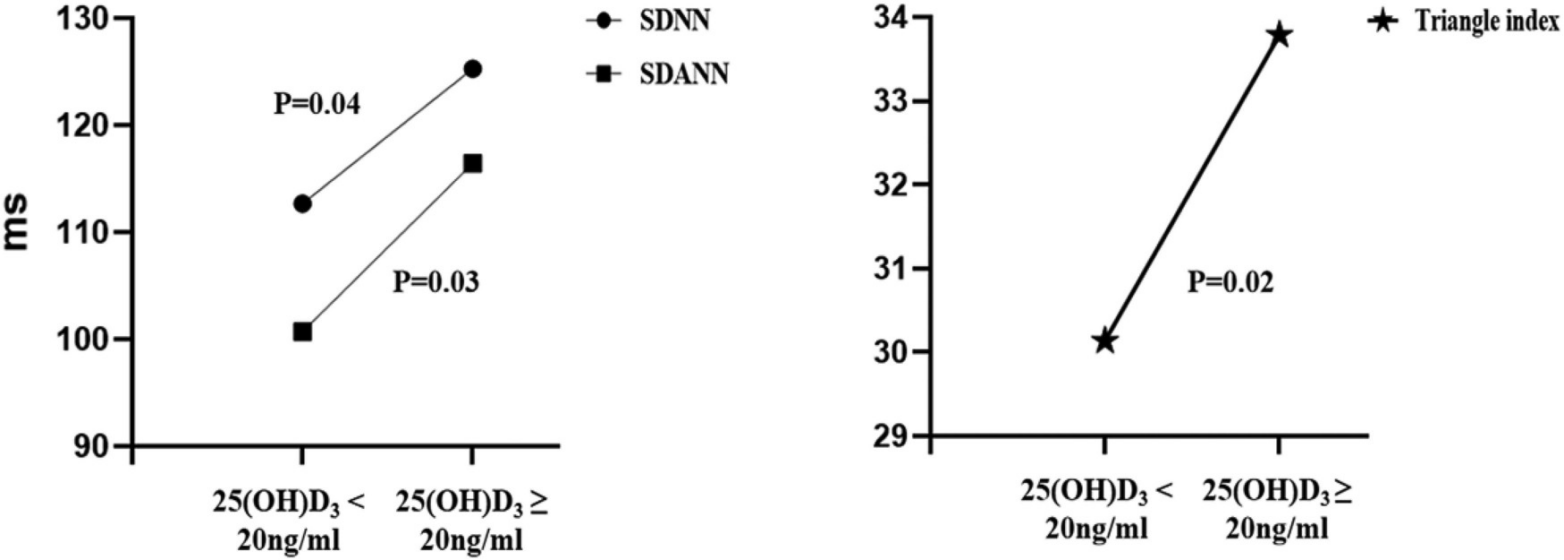
Comparison of different time domain HRV analysis parameters between the two groups. SDNN, standard deviation of all NN intervals; SDANN, standard deviation of the averages of NN intervals in all 5-min segments of the entire recording; Triangle index, Total number of all NN intervals divided by the height of the histogram of all NN intervals measured on a discrete scale with bins of 1/128 s.

Finally, 171(68.4%) patients had their 24-h Holter ECG monitoring completed in all study participants. There were 110 (64.3%) patients in the vitamin D deficiency group and 61 (35.7%) in the vitamin D non-deficiency group who had their 24-h Holter ECG monitoring completed. Table 3 shows the comparison of time-domain analysis values and frequency-domain analysis values obtained at baseline. In the time-domain analysis, hypertensive individuals with 25(OH)D_3_ < 20 ng/ml had lower SDNN, SDNN index, SDANN, RMSSD, PNN50, and Triangular Index than those with 25(OH)D_3_ ≥ 20 ng/ml (Table 3). And there was a difference between the SDNN values had statistically significant (112 ± 31 vs. 125 ± 41, *P* = 0.04), the difference is more pronounced in the SDANN (101 ± 28 vs. 117 ± 50, *P* = 0.03) and Triangle indexes (30 ± 9 vs. 34 ± 11, *P* = 0.02) (Table 3 and Figure 3). In the frequency-domain analysis, there were no differences in the study group for LF, HF, or LF/HF (Table 3).

**Table 3:** The distribution of HRV indices among participants.

Parameter	25(OH)D_3_ < 20 ng/ml (*n* = 110)	25(OH)D_3_ ≥ 20 ng/ml (*n* = 61)	*P*-value
Time domain, statistical measures			
SDNN, (mean ± SD) ms	113 ± 31	125 ± 41	**0** **.** **04**
SDNN index, (mean ± SD) ms	46 ± 20	51 ± 20	0.14
SDANN, (mean ± SD) ms	101 ± 28	117 ± 50	**0** **.** **03**
RMSSD, (mean ± SD) ms	29 ± 16	32 ± 33	0.38
PNN50, median (IQR) %	4.0 (1.4, 9.7)	4.6 (1.3, 8.8)	0.75
Triangle index	30 ± 9	34 ± 11	**0** **.** **02**
Frequency domain, long-term recordings (24 h)			
LF, median (IQR) ms^2^	270 (134, 460)	267 (167, 498)	0.29
HF, median (IQR) ms^2^	210 (122, 451)	236 (116, 447)	0.69
LF/HF	1.0 (0.7, 1.7)	1.12 (0.8, 1.8)	0.49

Abbreviations of HRV indices: HF, high-frequency band (in the 0.15–0.4 Hz frequency band); LF, low- frequency band (in the 0.04–0.15 Hz frequency band); PNN50, NN50 count divided by the total number of all NN intervals; RMSSD, the square root of the mean of the sum of the squares of differences between adjacent NN intervals; SDANN, standard deviation of the averages of NN intervals in all 5-min segments of the entire recording; SDNN, standard deviation of all NN intervals; SDNN index, Mean of the standard deviations of all NN intervals for all 5-min segments of the entire recording; Triangle index, Total number of all NN intervals divided by the height of the histogram of all NN intervals measured on a discrete scale with bins of 1/128 s.

A *P*-value of <0.05 indicates statistical significance.

### 3.4 The distribution of the aldosterone-to-renin ratio (ARR)

In both the supine and standing positions, vitamin D deficiency hypertensive patients had lower direct renin, aldosterone, and ARR values than those with vitamin D non-deficiency (Table 4). Hypertensive patients with vitamin D deficiency had a significantly greater aldosterone value than those having vitamin D non-deficiency. The median aldosterone value was 16 ng/dl (IQR: 10–18 ng/dl) for hypertensive individuals with 25(OH)D_3_ < 20 ng/ml vs. 12 ng/dl (IQR: 7–17 ng/dl) for hypertensive individuals with 25(OH)D_3_ ≥ 20 ng/ml (*P* = 0.02) in standing position, and the median aldosterone value was 10 ng/dl (IQR: 7–12 ng/dl) for hypertensive individuals with 25(OH)D_3_ < 20 ng/ml vs. 8 ng/dl (IQR: 6–12 ng/dl) for hypertensive individuals with 25(OH)D_3_ ≥ 20 ng/ml (*P* = 0.009) in the supine position. Similarly, ARR values were higher in the vitamin D deficiency group in both supine and standing positions. The median ARR value was 1.4 (IQR: 0.5–3.3) for hypertensive individuals with 25(OH)D_3_ < 20 ng/ml vs. 0.9 (IQR: 0.4–2.9) for hypertensive individuals with 25(OH)D_3_ ≥ 20 ng/ml (*P* = 0.03) in standing position, and the median ARR value was 1.9 (IQR: 0.6–4.1) for hypertensive individuals with 25(OH)D_3_ < 20 ng/ml vs. 1.1 (IQR: 0.5–3.4) for hypertensive individuals with 25(OH)D_3_ ≥ 20 ng/ml (*P* = 0.04) in the supine position (Table 4).

**Table 4:** The distribution of the aldosterone-to-renin ratio (ARR).

Renin and aldosterone levels	25(OH)D_3_ < 20 ng/ml (*n* = 165)	25(OH)D_3_ ≥ 20 ng/ml (*n* = 85)	*P*-value
Standing position			
Direct renin, median (IQR) μIU/ml	19 (6, 33)	16 (8, 33)	0.74
Aldosterone, median (IQR) ng/dl	16 (10, 18)	12 (7, 17)	**0** **.** **02**
ARR	1.4 (0.5,3.3)	0.9 (0.4, 2.9)	**0** **.** **03**
Supine position			
Direct renin, median (IQR) μIU/ml	10 (3, 21)	8 (4, 18)	0.78
Aldosterone, median (IQR) ng/dl	10 (7, 12)	8 (6, 12)	**0** **.** **009**
ARR	1.9 (0.6,4.1)	1.1 (0.5,3.4)	**0** **.** **04**

ARR, aldosterone to renin ratio.

A *P*-value of <0.05 indicates statistical significance.

## 4 Discussion

The main findings from this investigation were that vitamin D deficiency patients with hypertension had more severe coronary artery disease and more patients underwent coronary revascularization than vitamin D deficiency patients without hypertension. 25(OH)D_3_ < 20 ng/ml was an independent risk indicator for the occurrence of coronary revascularization in individuals with hypertension. And vitamin D deficiency affects HRV in hypertensive patients. We found that SDNN, SDANN, and triangular index were lower in participants with 25(OH)D_3_ deficiency. This implies that low serum 25(OH)D_3_ levels and cardiac autonomic dysfunction are related. Furthermore, Vitamin D deficiency affects ARR values in hypertensive patients.

Several studies have explored the correlation between vitamin D deficiency and coronary artery disease (CAD) (31–33). A meta-analysis including 19 prospective studies revealed an inverse relationship between the risk of CVD and 25(OH)D concentration between 20 and 60 mmol/L (34). Previous studies have found that Insufficient vitamin D levels were related to acute myocardial infarction in coronary heart disease (35). Low vitamin D levels are believed to be linked to both the risk of coronary artery disease and how serious the disease is. Furthermore, Vitamin D deficiency has already been coupled to severe coronary stenosis that is multivessel and more diverse in individuals who have undergone coronary angiography (36, 37). Consistent with this, our study found that despite no significant differences between the two groups, vitamin D deficiency hypertensive patients developed more coronary multi-vessel lesions than vitamin D non-deficiency hypertensive patients. And our results demonstrate that there was significantly more coronary revascularization in vitamin D deficiency hypertensive patients and associated with a more than two-fold higher risk of coronary revascularization compared with vitamin D non-deficiency patients.

The combined action of sympathetic and parasympathetic nerves determines the HRV, reduced HRV has not only been associated with different mental disorders and cognitive impairments but also with poor cardiovascular health (38–40). The SDNN is dependent on changes in all HRV parameters, reflecting the regulation of autonomic nerves on the body's heart rhythm and heart rate. Previous research has indicated that PNN50 and RMSSD are parasympathetic activity measures, whereas SDNN and SDANN represent sympathetic and parasympathetic heart rate modulation (26). A reduced HRV triangular index was a sign of autonomic nervous system imbalance and was a predictor of unfavorable outcomes such as malignant arrhythmias and mortality (41, 42). The decreased LF/HF ratio seems to be a distinctive indication of an imbalance among both sympathetic reflex interactions and the switch to sympathetic withdrawal, as well as the ensuing vagal emergence. Therefore, Low HRV values are generally indicative of sympathetic innervation, which could be attributed to sympathetic hyperactivity or parasympathetic hypoactivity (43). Few studies have investigated the effect of vitamin D levels on cardiac neurological function in hypertensive patients. In the current investigation, we discovered a correlation between circulating vitamin D levels and HRV in hypertensive patients, and we speculated that lower vitamin D levels in these patients may result in a lower HRV index. In animal studies, 1,25 (OH) _2_D was found to affect cardiac autonomic nervous activity (13, 44), which suggests that the deficiency of 1,25 (OH) _2_D results in abnormal cardiac functions. A cross-sectional study based on a Korean population found that lower 25(OH)D levels were associated with lower HRV (19). Consistent with this, our study found that SDNN, SDANN, and Triangle index were low in hypertensive individuals with 25(OH)D deficiency. Vitamin D has a direct effect on cardiomyocytes and also can affect cardiac contractility directly through vitamin D receptors (13). Our study found that hypertensive patients with vitamin D deficiency had low heart rate variability, therefore, low parasympathetic activity in hypertensive patients with vitamin D deficiency levels. Previous studies have found that heart dysfunction is significantly influenced by reduced parasympathetic tone (45). Therefore, 25(OH)D levels affect early pathophysiological changes in the heart and may cause cardiac dysfunction.

The important role of renin in the regulation of blood pressure changes is now well recognized. It was found that in VDR knock-out mice, renin expression was increased and the RAAS was activated, leading to increased blood pressure and cardiac hypertrophy (46). Thus, vitamin D deficiency could activate RAAS. According to our findings, aldosterone and ARR values were significantly higher in the vitamin D deficiency group compared to the vitamin D non-deficiency group, and there was demonstrated a statistically significant distinction between the two groups. Despite direct renin values being higher in the vitamin D deficiency group. This suggests that vitamin D deficiency affects renin and aldosterone expression in patients with hypertension, confirming the concept that vitamin D deficiency affects RAAS.

It has been demonstrated that cardiovascular risk factors including hypertension, diabetes, and dyslipidemia are related to deficiency of vitamin D (47). Large-scale studies have confirmed the connection between low levels of serum vitamin D and the development of CVD (48). Therefore, based on our research results, future vigilance is needed to prevent the development of coronary vascular disease, changes in cardiac autonomic function, and activation of RAAS in clinically hypertensive patients with vitamin D deficiency.

## 5 Study limitations

First, as a single-center observational study, the overall sample size of the study was small, and the current research may not be perfectly illustrative of all clinical practices. Further extensive investigations should be conducted to figure out the utility of vitamin D to reduce the incidence of cardiovascular disease and cardiac autonomic impairment. Second, an external examination of the research is required to validate whether managing vitamin D status can provide therapeutic assistance in clinical practice. Third, our study failed to follow up with patients to comprehend the effect of long-term vitamin D deficiency effects on cardiovascular disease events.

## 6 Conclusions

The present research point to a connection between vitamin D levels and the risk of coronary revascularization occurring. Our findings indicate that Vitamin D deficiency also affects HRV and ARR values in hypertensive patients. The findings indicate that vitamin D levels are critical to the development of CVD.

## Data Availability

The datasets presented in this study can be found in online repositories. The names of the repository/repositories and accession number(s) can be found in the article/Supplementary Material.
